# MET overexpression, gene amplification and relevant clinicopathological features in gastric adenocarcinoma

**DOI:** 10.18632/oncotarget.14382

**Published:** 2016-12-30

**Authors:** Jing Zhang, Lei Guo, Xiuyun Liu, Wenbin Li, Jianming Ying

**Affiliations:** ^1^ Department of Pathology, National Cancer Center/Cancer Hospital, Chinese Academy of Medical Sciences and Peking Union Medical College, Beijing, 100021, China

**Keywords:** gastric cancer, MET overexpression, MET gene amplification, biomarker, immunohistochemistry

## Abstract

This study was conducted to investigate the expression of MET in Chinese gastric adenocarcinoma cohort, the correlation between MET overexpression and clinical pathological features, HER2 expression and *MET* gene amplification. A total of 816 gastric adenocarcinoma patients were included and MET and HER2 immunohistochemical (IHC) staining were performed. IHC and dual-color silver in situ hybridization analysis were performed in the tissue microarrays, constructed from the 240 patients who were randomly selected. MET overexpression (IHC 3+) was observed in 6.0% (49/816) of the cohort. MET overexpression rate was higher in patients with poor prognostic factors, such as clinical stages III/IV (***p*** =0.012) and pathologic stages T3/T4 (***p*** =0.027). The HER2 overexpression (IHC 3+) rate was 8.8% (72/816) and MET overexpression rate was higher in HER2 positive patients (9.7%, 7/72). A high concordance rate (94.6%) between MET overexpression and gene amplification was demonstrated. Therefore, MET overexpression could serve as a prognostic biomarker and a potential therapeutic target for gastric cancer.

## INTRODUCTION

Gastric adenocarcinoma (GC) continues to be a significant public health threat. It is the fourth most common cancer and the second leading cause of cancer related death worldwide [1, 2]. The prognosis of patients with GC remains poor. The 5-year overall survival rate for advanced or metastatic disease remains <30% [3]. Trastuzumab, a recombinant monoclonal antibody targeting human epidermal growth factor receptor type 2 (HER2), is now being applied in GC treatment. It significantly improved the overall survival in patients with HER2 overexpression [4]. However, HER2 overexpression only occurs in approximately 13% of the Chinese GC patients [5]. Thus, more targeted agents for treating GC patients are in great needs.

Mesenchymal–epithelial transition factor (MET) is the so called hepatocyte growth factor (HGF) receptor. MET overexpression and its gene amplification have recently been indicated as survival prognostic factors for many cancers including GC. Multiple studies have shown that MET is also a potential therapeutic target in advanced or metastatic GC [6–8]. Interactive crosstalk between MET and other receptors such as epidermal growth factor receptor type 1 (EGFR) and HER2 underlies a key role in resistance to other Receptor Tyrosine Kinase-targeted therapies. For example, previous studies reported that MET pathways activation represented novel drug resistance mechanism of lapatinib unresponsiveness in HER2 positive gastric cancer [9, 10]. So combinational therapy may be more effective in treating HER2 positive gastric cancer patients with MET-induced resistance [11]. Patients with *MET* gene amplification showed transient sensitivity to the targeted MET inhibitor-Crizotinib [12, 13]. There was also case report about durable complete response of metastatic gastric cancer with anti-MET therapy [14]. Rilotumumab and Onartuzumab are both monoclonal antibodies that are MET inhibitors. Results from phase II clinical trials of these two MET inhibitors were encouraging [15] and currently there are multiple on-going phase III studies on these two agents and other MET inhibitors [16, 17].

Although MET targeted agents for gastric cancer have been intensively investigated, the categorizing the MET expression level into under/over expression is lacking standardized scoring system. Data from previous studies have shown MET overexpression rates vary from 4% to 82.4% [18, 19], and *MET* gene amplification rates vary from 2% to 24% [6], which may be the results of different patient populations and the lack of standardized scoring system used to categorize the expression level in these studies. In addition, the relationship between MET overexpression and *MET* gene amplification has rarely been studied. The primary objective of our study is to develop a modified score system for categorizing MET expression level using as standardized criteria, and assessed the incidence of MET overexpression in a large Chinese GC cohort. The relationship between MET overexpression and gene amplification and clinicopathological parameters including HER2 expression were also investigated in this study.

## RESULTS

### MET expression condition in chinese GC cohort without history of treatment

A total of 816 patients were evaluated by IHC. The patients’ clinicopathological characteristics were summarized in Table 1. The median age was 61 years (range from 23 to 84 years), with majority of male (73.9%). Of all patients, there were 318 cases (39%) of intestinal type by Lauren's classification. Approximately one-third was primary gastroesophageal junction (GEJ) adenocarcinoma and one-half was primary gastric body (GB) cancer. The tumors were poorly differentiated in 76.1%, moderately differentiated in 20.3%. There were 28.6% patients in clinical stage I, 19.9% in clinical stage II and 50.0%, 1.6% in stages III and IV. There were 23.7% patients at pathologic stage T1, with 12.3%, 29.2%, and 34.9% at stages T2, T3, and T4, respectively. About pathologic N stages, there were 35.3%, 17.2%, 18.6%, 28.9% patients at N0, N1, N2, and N3 stages respectively. Of the 816 patients, 49 (6.0%) patients were scored 3+ based on the modified scoring system, thus were considered MET overexpression positive (3+), while 767(94.0%) were identified as MET overexpression negative (with IHC score of 0/1+/2+). Heterogeneous overexpression of MET was found in 51% (25/49) MET IHC 3+ (overexpression) patients (Table 2).

**Table 1:** Correlation between MET expression status and GC clinicopathological parameters

IHC (%)	Groupingoverall	METNegative	METPositive	*P* value
**Pathologic features**	n=816(%)	n=767(94.0)	n=49(6.0)	
**Age at diagnosis**				0.769
**≥60 years**	448(54.9)	420(93.8)	28(6.3)	
**<60 years**	368(45.1)	347(94.3)	21(5.7)	
**Sex**				0.131
**Male**	603(73.9)	562(93.2)	41(6.8)	
**Female**	213(26.1)	205(96.2)	8(3.8)	
**Tumor location**				0.064
**GEJ**	253(31.0)	232(91.7)	21(8.3)	
**GB**	414(50.7)	397(95.9)	17(4.1)	
**GA**	149(18.3)	138(92.6)	11(7.4)	
**Lauren classification**				0.153
**Intestinal**	318(39.0)	294(92.5)	24(7.5)	
**Mixed**	233(28.6)	218(93.6)	15(6.4)	
**Diffuse**	265(32.5)	255(96.2)	10(3.8)	
**Tumor differentiation**				0.904
**Well**	29(3.6)	27(93.1)	2(6.9)	
**Moderately**	166(20.3)	155(93.4)	11(6.6)	
**Poorly**	621(76.1)	585(94.2)	36(5.8)	
**Clinical stages**				0.012
**I/II**	395(48.4)	380(96.2)	15(3.8)	
**III/IV**	421(51.6)	387(91.9)	34(8.1)	
**Clinical stages**				0.038
**I**	233(28.6)	233(95.7)	10(4.3)	
**II**	162(19.9)	157(96.9)	5(3.1)	
**III**	408(50.0)	374(91.7)	34(8.3)	
**IV**	13(1.6)	13(100)	0(0)	
**pT status**				0.021
**pT1/2**	293(35.9)	283(96.6)	10(3.4)	
**pT3/4**	523(64.1)	484(92.5)	39(7.5)	
**pT status**				0.027
**pT1**	193(23.7)	189(97.9)	4(2.1)	
**pT2**	100(12.3)	94(94.0)	6(6.0)	
**pT3**	238(29.2)	224(94.1)	14(5.9)	
**pT4**	285(34.9)	260(91.2)	25(8.8)	
**pN status**				0.644
**pN0**	288(35.3)	269(93.4)	19(6.6)	
**pN1/2/3**	528(64.7)	498(94.3)	30(5.7)	
**pN status**				0.801
**pN0**	288(35.3)	269(93.4)	19(6.6)	
**pN1**	140(17.2)	134(95.7)	6(4.3)	
**pN2**	152(18.6)	142(93.4)	10(6.6)	
**pN3**	236(28.9)	222(94.1)	14(5.9)	

**Table 2:** MET expression proportion in different expression intensity in GC

Expressionproportion	MET IHC			*P* value<0.001Total
	3+	2+	1+/0	
**≥50%**	24(49.0)	311(84.3)	301(75.6)	636(77.9)
**<50%**	25(51.0)	58(15.7)	97(24.4)	180(22.1)
**Total**	49(6.0)	369(45.2)	398(48.8)	816

### Relationship between MET overexpression and clinicopathological characteristics

Table 1 summarized MET overexpression status by subgroup of clinicopathological characteristics. Significant difference in MET overexpression was found in tumor clinical stage and pT status. MET overexpression was correlated with clinical stages with 8.1% (34/421) overexpression in III/IV stages patients and 3.8% (15/395) in I/II stages (***p*** =0.012). MET was overexpressed in 7.5%(39/484) of pT3/pT4 stage patients which is significantly higher than that in pT1/T2 stages (3.4%, 10/283). No correlation was observed between MET overexpression and age group, sex, tumor location, Lauren classification and tumor differentiation.

### MET expression and HER2 expression

The correlation between MET expression and HER2 expression in GC was summarized in Table 3. The positive rate for HER2 was 8.8% (72/816) and MET overexpression was higher in HER2 positive patients (9.7%, 7/72) than that in HER2 negative (5.1%, 32/622), although there was no significant difference (***p*** =0.164).

**Table 3:** Correlation between MET expression status and HER2 expression status in GC

HER2 (%)	MET		*P* value =0.164Total
	3+	0/1+/2+	
**0/1+**	32(5.1)	590(94.9)	622(76.2)
**2+**	10(8.2)	112(91.8)	122(15)
**3+**	7(9.7)	65(90.3)	72(8.8)
**Total**	49(6.0)	767(94)	816

### Concordance of MET overexpression and *MET* gene amplification

Dual-color silver in situ hybridization (DISH) analysis was performed in 240 GC and GEJ adenocarcinoma cases randomly selected, with high cell density (> 30% tumor cells/area) were selected. Finally, 205 fragments met the inclusion criteria for the study, including 16 cases with IHC 3+, 78 cases with IHC 2+, and 111 cases with IHC 0/1+. *MET* gene amplification was observed in 62.5% (10/16) of MET IHC 3+ cases, 2.6% (5/189) of MET IHC 2+/1+/0 cases, showing a concordance rate of 94.6% between IHC and DISH with a relatively low ***k*** value of 0.3 (Table 4).

**Table 4:** Correlation between MET overexpression and gene amplification in GC

*MET* DISH (%)	MET IHC			Total
	3+	2+	1+/0	
**MET/CEP7≥2**	10(62.5)	3(3.8)	2(1.8)	15(7.3)
**MET/CEP7<2**	6(37.5)	75(96.2)	109(98.2)	190(92.7)
**Total**	16(7.8)	78(38.1)	111(54.1)	205

## DISCUSSION

**Figure 1:**
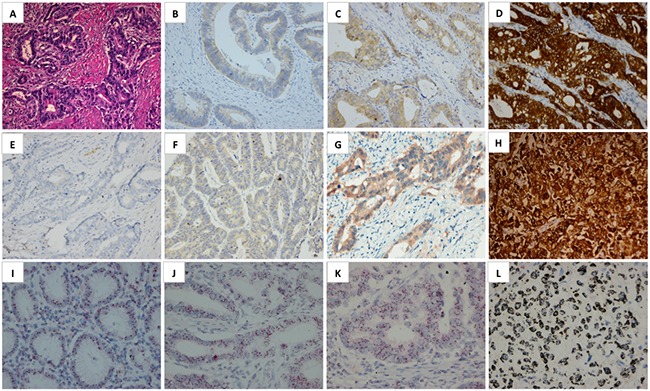
Representative microphotographs (A-H, ×200) of gastric cancer H&E staining A. and MET expression scored as 1+ B. 2+ C. 3+ D. in the membrane And MET expression interpreted as 0 **E**. 1+ **F**. 2+ **G**. and 3+ **H**. in both membrane and cytoplasm of tumor cells. Representative microphotographs of MET DISH (I-L, ×400): disomy of normal gastric musco (IHC -) **I**. IHC 1+ **J**. IHC 2+ **K**. and gene amplification **L**. cases by DISH are shown.

**Figure 2:**
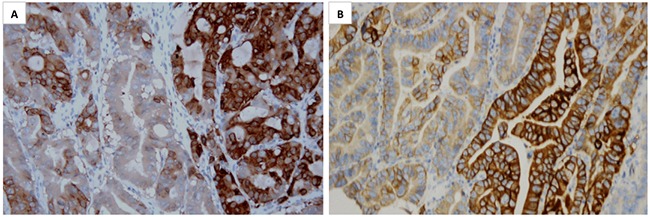
Representative microphotographs of MET overexpression heterogeneity are showed (A&B, ×200) Positive IHC staining of MET was seen in the right area but negative or weak staining in the left area in both picture **A**. and **B**.

Due to the inconsistent scoring criteria used in MET IHC interpretation in previous studies, in which the positivity rate of MET overexpression varying from 4% to 82.4% [18, 20, 21], this study demonstrated an applicable MET IHC scoring system in gastric cancer by combining expression proportion and intensity. Similar to Hofmann's criteria for HER2 IHC scoring [22], we chose 10% as a cut-off value which also has been used in other studies [23]. In the study of Sang Y Ha et al. strong staining in >10% of tumor cells was interpreted as MET IHC 3+ and all nine MET IHC 3+ cases showed MET gene amplification [24]. Our results showed that MET overexpression (IHC 3+) was detected in 6.0% (49/816) of the cohort. A high concordance rate (94.6%) between MET overexpression and gene amplification was demonstrated (Figure 1). MET expression heterogeneity was frequently found in MET overexpression cases, with 51% (25/49) MET IHC 3+ (overexpression) cases whose expression proportion were lower than 50%, while there were only 15.7% and 24.4% respectively in 2+ and 1+/0 patients (Table 2 and Figure 2). MET heterogeneity is definitely an issue that we should pay more attention to in our further studies.

Whether cases with MET IHC 2+ should be defined as MET overexpression is controversial. Some studies considered MET IHC 2+ as MET overexpression cases which is different from ours [25]. Previous study has shown that IHC 3+ and gene amplification were significantly associated with poor prognosis, but not for IHC 2+[26]. Meanwhile, in our study the MET IHC 2+ patients were rare with gene amplification (3.8%, 3/78). However, Sang Y Ha et al. also revealed MET IHC 2+ cases showed similarly poor survival as those with MET IHC 3+ cases, further confirmation should be performed [24].

The membranous and/or cytoplasmic expression levels of MET were considered while defining MET overexpression status. Jianji Jiang, et al have indicated that only simultaneous membranous and cytoplasmic overexpression of MET was significantly correlated with *MET* gene amplification and showed shorter overall survival and disease-free survival compared with patients without MET overexpression [27]. In our study MET overexpression status defined solely base upon membranous level, cytoplasm level or simultaneous membranous and cytoplasmic level was not found to be correlated with any clinicopathological characteristics.

Other semi-quantitative methods for evaluating MET IHC staining were also explored. In the study of Tiankang Guo et al., proportional score (0, 0%; 1, 1-25%; 2, 26%-50%; 3, 51%-75% and 4, 76-100%) and intensity score (0, negative; 1, weak; 2, medium and 3, strong) were summed to obtain a total score. A score ≥ 3 was considered as positive expression [28]. They reported the positive rate of MET was 59.18% and expression of MET protein was significant correlated with lymph node metastasis (***p*** = 0.041). We applied the method in our study and summarized the result in Table 5. MET positive rate was 87.9% and although expression of MET protein was significant correlated with lymph node metastasis (***p*** < 0.001), MET positive was higher in pN0 stage. We had contradictory conclusion with a large sample, so this method maybe not scientific and maybe IHC intensity was the only factor need to be considered in MET IHC scoring system.

**Table 5:** Correlation between MET expression status and GC clinicopathological parameters (by applying the semi-quantitative methods for evaluating MET IHC staining of the study of Tiankang Guo et al.)

IHC (%)	Grouping overall	MET Negative	MET Positive	*P* value
**Pathologic features**	n=816(%)	n=99(12.1)	n=717(87.9)	
**Age at diagnosis**				0.162
**≥60 years**	448(54.9)	61(13.6)	387(86.4)	
**<60 years**	368(45.1)	38(10.3)	330(89.7)	
**Sex**				0.066
**Male**	603(73.9)	81(13.4)	522(86.6)	
**Female**	213(26.1)	18(8.5)	195(91.5)	
**Tumor location**				0.008
**GEJ**	253(31.0)	43(17.0)	210(83.0)	
**GB**	414(50.7)	37(8.9)	377(91.1)	
**GA**	149(18.3)	19(12.8)	130(87.2)	
**Lauren classification**				0.127
**Intestinal**	318(39.0)	33(10.4)	285(89.6)	
**Mixed**	233(28.6)	25(10.7)	208(89.3)	
**Diffuse**	265(32.5)	41(15.5)	224(84.5)	
**Tumor differentiation**				0.324
**Well**	29(3.6)	6(20.7)	23(79.3)	
**Moderately**	166(20.3)	18(10.8)	148(89.2)	
**Poorly**	621(76.1)	75(12.1)	546(87.9)	
**Clinical stages**				0.107
**I/II**	395(48.4)	40(10.1)	355(89.9)	
**III/IV**	421(51.6)	59(14.0)	362(86.0)	
**Clinical stages**				0.006
**I**	233(28.6)	14(6.0)	219(94.0)	
**II**	162(19.9)	26(16.0)	136(84.0)	
**III**	408(50.0)	58(14.2)	350(85.8)	
**IV**	13(1.6)	1(7.7)	12(92.3)	
**pT status**				0
**pT1**	193(23.7)	10(5.2)	183(94.8)	
**pT2**	100(12.3)	17(17)	83(83)	
**pT3**	238(29.2)	42(17.6)	196(82.4)	
**pT4**	285(34.9)	30(10.5)	255(89.5)	
**pT status**				0.058
**pT1/2**	293(35.9)	27(9.2)	266(90.8)	
**pT3/4**	523(64.1)	72(13.8)	451(86.2)	
**pN status**				0
**pN0**	288(35.3)	19(6.6)	269(93.4)	
**pN1/2/3**	528(64.7)	80(15.2)	448(84.8)	
**pN status**				0.003
**pN0**	288(35.3)	19(6.6)	269(93.4)	
**pN1**	140(17.2)	19(13.6)	121(86.4)	
**pN2**	152(18.6)	26(17.1)	126(82.9)	
**pN3**	236(28.9)	35(14.8)	201(85.2)	

We also explored the modified H-score system mentioned in the review of Luigi Marano et al. [23]. The analysis result was summarized in Table 6. MET positive rate was 42.0%. Significant difference in MET overexpression was found in tumor location, Lauren classification, clinical stages, pT status and pN status. MET overexpression was significant higher in I/II stage, pT1/2 and pN0 cases. We would draw a contrary conclusion to the previous studies mentioned in this review and other systematic review and meta-analysis [29].

**Table 6:** Correlation between MET expression status and GC clinicopathological parameters (by applying the modified H-score system for evaluating MET IHC staining mentioned in the review of Luigi Marano et al.)

IHC (%)	Grouping overall	MET Negative	MET Positive	*P* value
**Pathologic features**	n=816(%)	n=473(58.0)	n=343(42.0)	
**Age at diagnosis**				0.033
**≥60 years**	448(54.9)	275(61.4)	173(38.6)	
**<60 years**	368(45.1)	198(53.8)	170(46.2)	
**Sex**				0.419
**Male**	603(73.9)	355(58.9)	248(41.1)	
**Female**	213(26.1)	118(55.4)	95(44.6)	
**Tumor location**				0.002
**GEJ**	253(31.0)	170(67.2)	83(32.8)	
**GB**	414(50.7)	223(53.9)	191(46.1)	
**GA**	149(18.3)	80(53.7)	69(46.3)	
**Lauren classification**				0.008
**Intestinal**	318(39.0)	164(51.6)	154(48.4)	
**Mixed**	233(28.6)	139(59.7)	94(40.3)	
**Diffuse**	265(32.5)	170(64.2)	95(35.8)	
**Tumor differentiation**				0.169
**Well**	29(3.6)	12(41.4)	17(58.6)	
**Moderately**	166(20.3)	95(57.2)	71(42.8)	
**Poorly**	621(76.1)	366(58.9)	255(41.1)	
**Clinical stages**				0
**I/II**	395(48.4)	200(50.6)	195(49.4)	
**III/IV**	421(51.6)	273(64.8)	148(35.2)	
**Clinical stages**				0
**I**	233(28.6)	107(45.9)	126(54.1)	
**II**	162(19.9)	93(57.4)	69(42.6)	
**III**	408(50.0)	264(64.7)	144(35.3)	
**IV**	13(1.6)	9(69.2)	4(30.8)	
**pT status**				0
**pT1/2**	293(35.9)	137(46.8)	156(53.2)	
**pT3/4**	523(64.1)	336(64.2)	187(35.8)	
**pT status**				0
**pT1**	193(23.7)	84(43.5)	109(56.5)	
**pT2**	100(12.3)	53(53.0)	47(47.0)	
**pT3**	238(29.2)	156(65.5)	82(34.5)	
**pT4**	285(34.9)	18.(63.2)	105(36.8)	
**pN status**				0
**pN0**	288(35.3)	142(49.3)	146(50.7)	
**pN1/2/3**	528(64.7)	331(62.7)	197(37.3)	
**pN status**				0
**pN0**	288(35.3)	142(49.3)	146(50.7)	
**pN1**	140(17.2)	77(55.0)	63(45.0)	
**pN2**	152(18.6)	102(67.1)	50(32.9)	
**pN3**	236(28.9)	152(64.4)	84(35.6)	

Previous studies have indicated MET overexpression activated MET signal pathway to promote tumor cell growth, survival, migration, and invasion as well as tumor angiogenesis [19, 26, 30, 31]. In our study, one of the largest studies to date, MET overexpression was found to be related to clinical stages and pT stages. Advanced pT stages supported MET proto-oncogene activation for deeply infiltrating in gastric cancers. It indicated that MET overexpression was poor prognostic factor. That is consistent with many other reports [21, 25, 29]. One study has reported that overexpression of MET tended to be associated with poor prognosis, but there were no significant effects after adjustment for potentially confounding factors with multivariate analysis [32]. Definitely this speculation should be further validated by directly analysis the correlation between MET overexpression and GC overall survival or disease-free survival.

The present study retrospectively evaluated the correlation of MET expression and HER2 expression. According to Table 2, although there was no significant correlation among the subgroups (***p*** =0.164), we found that MET overexpression rate was higher in HER2 positive patients (9.7%, 7/72). Similarly, other researchers revealed that the HER2-positive rate was significantly higher in MET–positive tumors than that in MET–negative tumors (***p*** =0.036) [32]. Meanwhile previous study also reported that co-overexpression of MET and HER2 was demonstrated in small subsets of GC (22%) associated with aggressive behavior. In these cases, combination therapy may be considered as a potential treatment option [11].

In this study, the concordance rate between MET expression and gene amplification was 94.6%. Six cases with MET IHC 3+ didn't show gene amplification. These results suggest that, instead of *MET* gene amplification, other mechanisms such as mutation or alternative splicing were responsible for MET protein overexpression. Five cases with *MET*/CEP 7 ratios ≥2 showed MET expression with IHC 2+, and they were low ratios with *MET*/CEP 7 ranging from 2.01 to 2.46. Due to the limitation of the sample size, the relationship between MET overexpression and *MET* gene amplification and the definition of *MET* gene amplification should be further investigated in future studies with larger sample sizes.

In summary, there were 6.0% (49/816) cases showing MET overexpression (IHC 3+) in this Chinese cohort of Chinese GC without history of treatment. MET overexpression (IHC 3+) was more common in cases with deeper infiltrating tumor and advanced clinical stages. *MET* gene amplification was the main reason for MET overexpression. MET overexpression could serve as a prognostic biomarker and a potential therapeutic target for gastric cancer.

## MATERIALS AND METHODS

### Samples

A total of 1206 patients with primary GC, who were diagnosed by postoperative pathology, between January 2014 and January 2015, at the Department of Pathology, Cancer Hospital, Chinese Academy of Medical Sciences, Beijing, China, were screened in this retrospective study. In total, 816 patients without history of neoadjuvant chemotherapy, radiotherapy and endoscopic submucous resection were included in this study. All tumor samples were fixed in 10% neutral buffered formalin for 24-48 hours and embedded in paraffin. MET and HER2 IHC staining were routinely performed in all tumor samples, and the MET, HER2 IHC slides and the relevant hematoxylin and eosin (H&E) staining slides were assessed independently by two pathologists. Clinicopathological parameters including age at diagnosis, sex, tumor localization, histological classification, Lauren's classification, pathological TNM stage and clinical stages were also recorded.

### Tissue microarrays

A total of 240 formalin-fixed paraffin-embedded samples were randomly selected to construct tissue microarrays (TMA). Two tumor cores of 1.0 mm diameter were taken from each sample based on H&E staining for conducting IHC staining and dual-color silver in situ hybridization (DISH) analysis.

### Immunohistochemistry

Expression of MET was evaluated by IHC analysis using rabbit monoclonal primary antibody against MET (SP44; Ventana, Tucson Arizona). Automated IHC was performed on 4-μm-thick sections using the Ventana Benchmark ULTRA automated slide processing system according to the manufacturer's instructions. A 4-step scoring system was used as follows: no cell membrane and/or cytoplasm staining or cell membrane and/or cytoplasm staining in < 10% of tumour cells (score 0), faint/barely perceptible partial cell membrane and/or cytoplasm staining in > 10% of tumour cells (score 1+), weak-to-moderate staining of the entire cell membrane and/or cytoplasm in > 10% of tumour cells (score 2+), and strong staining of the entire cell membrane and/or cytoplasm in > 10% of tumour cells (score 3+). MET overexpression negative is defined for patient with IHC staining scores of 0, 1+ and 2+, and positive if IHC staining scores of 3+ (Figure 1). Signal localization (cell cytoplasm or membrane) was also recorded for MET positive cases. We use Hofmann's criteria for HER2 IHC scoring [22]. TMA cores’ IHC scores were evaluated based on staining intensity (0, no staining; 1+, faint staining; 2+, weak or moderate staining; 3+, strong staining) and the stronger intensity of the two scores is the final IHC result of the sample.

### Dual-color silver in situ hybridization

Dual-color silver in situ hybridization (DISH) analysis was carried out with the *MET* DNA probe kit and procedures (Ventana). The kit contains dual-color-labeled DNA probes, *MET* gene (labeled with Spectrum Black) and CEP 7 (chromosome 7 enumeration probe, labeled with Spectrum Red). Pretreatment was carried out with the Paraffin Pretreatment Kit. The *MET* signals and CEP 7 signals of 20-40 nuclei of gastric tumor cells in different areas were counted using a LEICA DM3000 light microscope ×400. The *MET*/CEP 7 ratios were interpreted as follows: a *MET*/CEP 7 ratios≥2 was defined as amplification of the *MET* gene, while a ratio lower than 2 was defined as no amplification of the *MET* gene (Figure 2).

Both the IHC scoring and DISH analysis were performed microscopically by two independent pathologists who were blinded to the clinical characteristics of each individual patients. The case-by-case final consensus result was discussed and determined in a common session.

### Statistic analysis

Statistical analysis was carried out using SPSS 21.0 statistical software (SPSS, Inc., Chicago, IL). Statistical associations between clinicopathological characteristics and MET status were assessed using the χ2 test or Fisher's exact test (if subgroup figure is below expectation). All tests were two-sided, with significance level of 0.05. Kappa analysis was also conducted to assess the concordance of MET overexpression and MET gene amplification.

## Abbreviations

MET: mesenchymal–epithelial transition factor
GC: gastric adenocarcinoma
IHC: immunohistochemistry
DISH: dual-color silver in situ hybridization
HER2: human epidermal growth factor receptor type 2
GEJ: gastroesophageal junction
GB: gastric body
GA: gastric antrum
TNM: tumor, node and metastasis
TMA: Tissue Microarray.
